# Anxiety Sensitivity Domains are Differently Affected by Social and Non-social Autistic Traits

**DOI:** 10.1007/s10803-021-05228-w

**Published:** 2021-08-10

**Authors:** Chiara Baiano, Gennaro Raimo, Isa Zappullo, Roberta Cecere, Barbara Rauso, Monica Positano, Massimiliano Conson, The LabNPEE Group, Lea Dell’ Aversana, Alessandro Di Rosa, Giovanna Esposito, Rosa Milo, Francesco Polito, Camilla Raimondo, Agnese Turco

**Affiliations:** Department of Psychology, University of Campania Luigi Vanvitelli, Viale Ellittico 31, 81100 Caserta, Italy

**Keywords:** Anxiety sensitivity, Autistic traits, Depression, Sex differences, Cognitive concerns, Social concerns

## Abstract

Anxiety sensitivity (AS) is implicated in the development and maintenance of several psychopathological conditions. Non-clinical individuals with high autistic traits may develop anxiety disorders and depressive symptoms. Here, we investigated the relationships of autistic traits with AS dimensions and depression, considering sex. We referred to the two-factor model of the autism spectrum quotient to distinguish social and non-social autistic traits and assessed 345 university students on AS and depression scales. Results showed that only social autistic traits predicted general AS and anxiety-related concerns regarding social and cognitive domains. The present results emphasize the need of assessing multiple domains of anxiety in individuals on the autistic spectrum, differentiating social and non-social traits.

## Introduction

Anxiety sensitivity (AS) refers to fear of anxiety-related sensations, such as increased heartbeat or breathing, feeling dizzy or faint, coming from erroneous beliefs that these sensations will produce harmful consequences (Reiss & McNally, 1985). AS has been analysed in the light of three main domains (Taylor et al., 2007): (i) physical anxiety, referring to fear of somatic anxiety symptoms, which are believed to lead to catastrophic physical issues; (ii) social anxiety, defined as the belief that a public exhibition of anxiety symptoms will result in social rejection or derision; (iii) cognitive anxiety, referring to fear of the mental correlates of anxiety symptoms that are believed to signal a mental health disorder.

Available literature underscores the role of AS in the onset and the maintenance of several anxiety disorders. For instance, high AS levels have been reported in patients with panic disorder and agoraphobia (Reiss & McNally, 1985; White et al., 2006), social and specific phobias (Norton et al., 1997; Sandin Chorot & McNally, 1996), as well as obsessive–compulsive disorder (Calamari et al., 2008).

It has been suggested that AS intensifies anxiety, as highly anxiety-sensitive people find their own arousal to be dangerous, experiencing increased anxiety in response to fear-eliciting stimuli (Taylor, 1999). Hence, investigating AS in individuals who are prone to develop anxiety symptoms by assessing a set of domain-specific anxiety-related concerns rather than a unitary anxiety-proneness variable has important clinical implications for treatment (Telch et al., 1989).

Anxiety commonly occurs among individuals on the autism spectrum. Indeed, anxiety disorders comorbidity in autism spectrum conditions (ASC) has been reported in approximately 40% of individuals (Ketelaars et al., 2008; Meyer et al., 2006; Shtayermman, 2007; van Steensel et al., 2011). Although it is common for people with ASC to experience the same psychological and physical characteristics associated with anxiety in the general population, individuals with ASC also frequently show increased social anxiety, panic attacks and obsessive–compulsive disorders (Bellini, 2004; Postorino et al., 2017; van Steensel & Heeman, 2017). Non-clinical individuals with high autistic traits (Baron-Cohen et al., 2001) are more prone to develop emotional disorders as well. In particular, both university students and older adults with high autistic traits report more anxious and depressive symptoms than people with low autistic traits (Kanne et al., 2009; Scherff et al., 2014; Wainer et al., 2013; Wallace et al., 2016). Also, social anxiety can be predicted by autistic traits in university students (Dickter et al., 2018; Freeth et al., 2013). Accordingly, children with high autistic traits present more emotional problems than those with low autistic traits (Posserud et al., 2018; Tick et al., 2016). An association between autistic traits and severity of anxiety and depressive symptoms has been found (Pine et al., 2008; van Steensel et al., 2013), together with an increase of depressive symptoms across childhood and adolescence (Rai et al., 2018). Moreover, anxious adolescents with higher levels of non-clinical autistic traits are more likely to be diagnosed with anxiety disorders and are more resistant to traditional cognitive behavioural interventions for anxiety with respect to clinically anxious adolescents with lower autistic traits (Puleo & Kendall, 2011; Settipani & Kendall, 2013).

In synthesis, reviewed evidence indicates that individuals with high autistic traits are more likely to experience anxiety and depression. For this reason, investigating whether different autistic traits could be linked to specific psychopathological signs would allow to identify individuals who are at increased risk to develop clinically relevant emotional disorders, thus providing a heuristic for tailoring early treatment strategies.

Instruments such as the anxiety sensitivity index-3 (ASI-3,Petrocchi et al., 2014; Taylor et al., 2007) provides a valuable and reliable method for assessing different kinds of appraisals of anxiety consequences. In the present study, we administered the ASI-3 to a large sample of university students who were assessed on their autistic traits through the autism spectrum quotient (AQ; Baron-Cohen et al., 2001). Relevant here, growing data are revealing dissociations between social and non-social domains both in clinical autism and typical development (Dworzynski et al., 2009; Greenberg et al., 2018; Grove et al., 2013; Palmer et al., 2015; Warrier et al., 2019), strengthening the view that a continuity exists across clinical autism and autistic traits in the general population (Baron-Cohen et al., 2001; Constantino & Todd, 2003; Robinson et al., 2011).

By capitalizing on these findings, here we used the two-factor model of AQ differentiating social traits (“Social interaction”) and non-social traits (“Attention to detail”) in neurotypicals (Hoekstra et al., 2008) to evaluate whether persons differing on these two dimensions of the spectrum would present specific cognitive, social or physical anxiety-related concerns, and depression. Indeed, as recalled above, due to large cooccurrence of anxiety and depression symptoms in persons with both clinical and non-clinical autism, a formalized assessment of depression was also performed by the Beck Depression Inventory-II (BDI-II, Beck, 1996; Sica & Ghisi, 2007). Finally, we evaluated whether the relationship between social and non-social autistic traits, AS and depression could be moderated by sex. Sex differences have been largely demonstrated in autistic traits, with men showing higher traits than women (Baron-Cohen et al., 2003; Greenberg et al., 2018), and recent data revealed a moderating effect of sex on the relationship between autistic traits and psychopathological behaviours (Barnett et al., 2021). Sex differences in AS are more debated both in clinical and non-clinical samples, with some authors reporting higher AS in women than men, while others failing to detect sex differences in AS levels (Escocard et al., 2009; Jurin et al., 2012; Osman et al., 2010; Petrocchi et al., 2014; Taylor et al., 2007). For these reasons, we also aimed at verifying whether sex could interact with social and non-social traits in predicting AS and depressive symptoms.

## Methods

### Participants

For the present study, participants were 345 university students (241 females and 104 males) recruited from different universities in the Campania region, Southern Italy. Participants studied at their universities for at least one year (mean = 4.51; SD = 1.14; range = 1–6 years). All participants spoke Italian as their native language, had a mean age of 23.63 years (SD = 2.85; range: 18–34; females: mean age = 23.22 years; SD = 2.76; males: mean age = 24.59 years; SD = 2.86).

To be included in the study, each participant had to meet the following selection criteria: (i) lack of any neurological or neurodevelopmental condition; (ii) lack of any history of actual clinically relevant psychopathological conditions.

The research was conducted after participants provided written informed consent approved by the Local Ethics Committee and performed in accordance with the ethical standards laid down in the 1964 Declaration of Helsinki.

### Measures

Participants underwent three psychometrically valid self-report questionnaires assessing autistic traits, AS and depression. The assessment was performed through a specific online platform (Google Forms, Google Inc., Mountain View, CA, USA).

#### Autistic Traits

The Autism Spectrum Quotient (AQ; Baron-Cohen et al., 2001; Ruta et al., 2012) quantifies autistic traits across five domains (social skill, attention switching, attention to detail, communication, and imagination) in both clinical and non-clinical samples. Participants underwent the full 50-item AQ with higher scores indicating higher autistic traits (Baron-Cohen et al., 2001; range score 0–50). Results were scored according to Baron-Cohen et al. (2001) criteria, resulting in a total AQ score and in further five scores for the corresponding five subscales: social skill, attention switching, attention to detail, communication and imagination. The Italian version of the AQ showed a fair internal consistency (Cronbach’s α: AQ total = 0.74; communication = 0.62; social skill = 0.65; imagination = 0.54; attention to details = 0.61, attention switching = 0.57) in neurotypical subjects and a good 6-months test–retest reliability (Pearson *r* = 0.79; Ruta et al., 2012).

To measure social and non-social related autistic traits the two-factor model of the autism spectrum quotient (AQ; Baron-Cohen et al., 2001) was adopted. In detail, we separate a “Social interaction” factor (i.e., AQ social traits) and “Attention to detail” factor (i.e., AQ non-social traits) with higher scores indicating respectively greater social difficulties and greater attention to details (Hoekstra et al., 2008).

Following the two-factor model of the autism spectrum quotient (AQ; Baron-Cohen et al., 2001) proposed by Hoekstra et al. (2008), here a confirmatory factor analysis was performed on AQ social skills, AQ attention switching, AQ communication, AQ imagination and AQ attention to detail subscales scores. Results of the principal component analysis confirmed the presence of one higher order factor, existing of four lower order domains (Standardized saturations on the first factor: AQ social skills = 0.736; AQ attention switching = 0.664; AQ communication = 0.754; AQ imagination = 0.499; variance explained: 36.19; eigenvalues: 1.810) and of one separate factor consisting in AQ attention to detail (Standardized saturations on the second factor: AQ attention to detail = 0.980; variance explained: 20.04; eigenvalues: 1.002). Therefore, the AQ social traits variable was computed through the sum of the for sub-scales (i.e., AQ social skills, AQ attention switching, AQ communication, AQ imagination). The non-social domain (AQ non-social traits) consisted of AQ attention to detail subscale raw score (Hoekstra et al., 2008). For both factors, higher scores correspond to stronger autistic traits; namely, higher scores imply greater social difficulties and greater attention to details, respectively.

#### Anxiety Sensitivity

The Anxiety Sensitivity Index-3 (ASI-3, Petrocchi et al., 2014; Taylor et al., 2007) is a self-report scale that measures the degree of concerning about possible negative consequences of anxiety symptoms. The ASI-3 provides three factors: (i) ASI-3 physical concerns as the fear of somatic anxiety symptoms, which are believed to lead to a catastrophic physical issue; (ii) ASI-3 social concerns as the belief that a public exhibition of anxiety symptoms will result in public; (iii) ASI-3 cognitive concerns as the fear of the mental correlates of anxiety symptoms, considered as signals of a mental disorder. The ASI-3 includes 18 items and a total score ranging from 0 to 72, with higher scores indicating higher AS levels. The Italian version of the ASI-3 showed a good internal consistency (Cronbach’s α: ASI-3 total score = 0.90; physical concerns = 0.87; social concerns = 0.81; cognitive concerns = 0.83) in non-clinical individuals (Petrocchi et al., 2014).

#### Depressive Symptoms

The Beck depression inventory-II (BDI-II, Beck, 1996; Sica & Ghisi, 2007) is one of the most widely used psychometric tests for the assessment of depression severity. It consists of 21 items investigating depressive symptoms, as sense of failure, guilt, social withdrawal, insomnia, or weight loss. The total score ranges from 0 to 63, with higher scores reflecting higher levels of depression. For the Italian version of the scale, internal consistency was α = 0.82 for the non-clinical sample, and α = 0.89 for the clinical sample (Sica & Ghisi, 2007).

### Statistical Analysis

Preliminary descriptive analyses were executed on AQ total scores and subscales for the whole group and for females and males separately.

Then, multiple regressions were conducted to investigate whether social and non-social autistic traits (i.e., AQ social and AQ non-social) and sex specifically predicted AS and depression. We also investigated the possible interaction between sex and social and non-social autistic traits in the prediction of AS and depression by computing interaction variables (i.e., Sex × AQ social traits; Sex × AQ non-social traits; AQ social traits × AQ non-social traits; Sex x AQ social traits × AQ non-social traits). Four linear multiple regressions were conducted by entering AQ social, AQ non-social traits, sex, and their interactions as independent variables. AS domains (i.e., ASI-3 social, ASI-3 cognitive, ASI-3 physical concerns scores and ASI-3 total score) and depression (i.e., BDI-II total score) were entered as dependent variables. In each regression model, all variables were included as z-scores while sex that was dummy coded (males = 0; females = 1). All the analyses were performed up using the Statistical Package for Social Sciences (SPSS Inc, version 25).

## Results

AQ total scores and subscales for the whole group and separately for sex are provided in Table 1. AS domains (ASI-3 total, social, physical and cognitive concern scores) and depression (BDI-II) scores for the entire group and separately for sex are reported in Table 2.

**Table 1:** Descriptive analysis of AQ total scores and subscales for the whole group and separately for sex

	Males (N = 104)	Females (N = 241)	Total (N = 345)
AQ total score	17.51 ± 5.00	16.42 ± 5.15	16.75 ± 5.12
AQ social traits	11.99 ± 4.56	10.91 ± 4.49	11.24 ± 4.53
Social skills	1.96 ± 1.87	1.90 ± 1.59	1.92 ± 1.68
Attention switching	4.78 ± 1.72	4.51 ± 1.91	4.59 ± 1.86
Communication	2.25 ± 1.72	2.23 ± 1.60	2.23 ± 1.64
Imagination	3 ± 1.75	2.28 ± 1.47	2.50 ± 1.59
AQ non-social traits (Attention to detail)	5.52 ± 2.17	5.51 ± 2.21	5.51 ± 2.20

The values are expressed as mean ± standard deviations

*AQ* autism spectrum quotient

**Table 2:** Descriptive analysis of anxiety sensitivity domains and depression scores for the whole group and separately for sex

	Males (N = 104)	Females (N = 241)	Total (N = 345)
ASI-3			
Total score	17.71 ± 11.75	20.30 ± 12.76	19.52 ± 12.51
Cognitive concerns	4.43 ± 4.44	5.33 ± 4.85	5.06 ± 4.74
Physical concerns	5.07 ± 5.15	6.68 ± 5.74	6.20 ± 5.61
Social concerns	8.21 ± 5.14	8.29 ± 5.01	8.26 ± 5.04
BDI-II total score	10.77 ± 8.06	12.18 ± 8.20	11.76 ± 8.17

The values are expressed as mean ± standard deviations

*ASI-3* anxiety sensitivity index, *BDI-II* Beck depression inventory, *AQ* autism spectrum quotient

### Regression Analyses

**Fig. 1:**
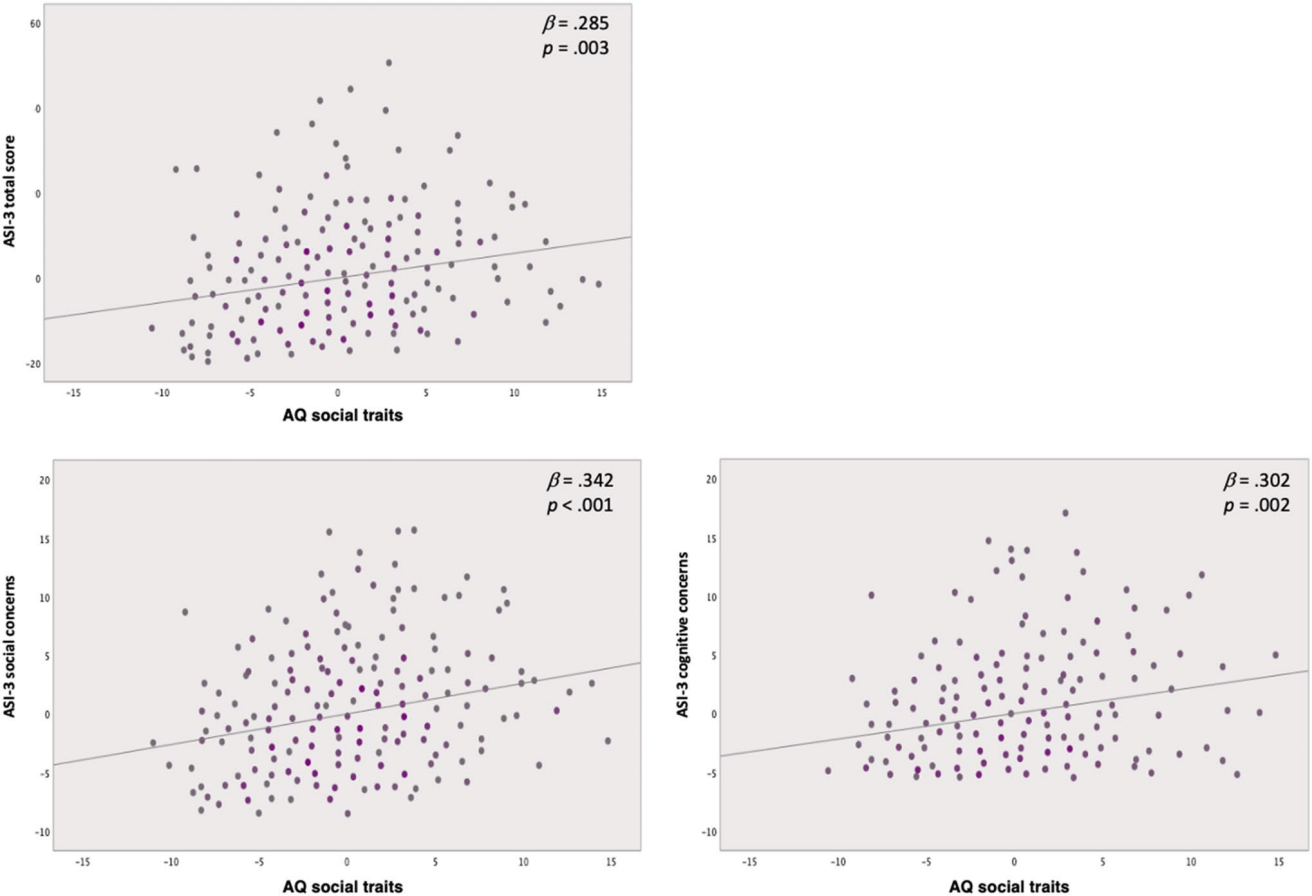
Top left panel: Regression plots of ASI-3 total score with AQ social traits. Bottom panels: Regression plots of ASI-3 social concerns with AQ social traits (left panel) and of ASI-3 cognitive concerns with AQ social traits (right panel). Grey dots represent females, purple dots are for males. *AQ* Autism Spectrum Quotient, *ASI-3* Anxiety Sensitivity Index

Data from multiple linear regression analyses (Table 3; Fig. 1) showed that the regression model for the prediction of AS assessed by ASI-3 total score was significant, *F*(7,341) = 3.35; *p* = 0.002; *R* = 0.255; *R*^*2*^ = 0.065. Results showed that AQ social traits (*β* = 0.285; *t* = 2.97; *p* = 0.003) and sex (*β* = 0.121; *t* = 2.27; *p* = 0.024) were two specific predictors of the measure.

**Table 3:** Multiple linear regression analyses predicting anxiety sensitivity and depression from sex, the AQ social and non-social traits and their interactions

	ASI-3 total score			ASI-3 cognitive concerns			ASI-3 physical concerns			ASI-3 social concerns			BDI-II total score		
Predictor	*R*^*2*^	*b* [95% *CI*]	*β*	*R*^*2*^	*b* [95% *CI*]	*β*	*R*	*b* [95% *CI*]	*β*	*R*^*2*^	*b* [95% *CI*]	*β*	*R*^*2*^	*b* [95% *CI*]	*β*
Fit model	0.255**			0.262**			0.179			0.253**			0.301***		
Sex		0.264 [0.036, 0.491]	0.121*		0.249 [0.021, 0.476]	0.114*		0.304 [0.072, 0.537]	0.140*		0.081 [− 0.147, 0.310]	0.037		0.219 [− 0.006, 0.444]	0.101
AQ social traits		0.284 [0.096, 0.472]	0.285**		0.302 [0.114, 0.490]	0.302**		0.071 [− 0.120, 0.263]	0.071		0.342 [0.153, 0.530]	0.342***		0.130 [− 0.056, 0.315]	0.130
AQ non-social traits		− 0.086 [− 0.284, 0.112]	− 0.087		− 0.104 [− 0.302, 0.094]	− 0.104		− 0.060 [− 0.262, 0.142]	− 0.06		− 0.049 [− 0.248, 0.149]	− 0.050		0.090 [− 0.105, 0.286]	0.091
Sex × AQ social traits		− 0.081 [− 0.309, 0.147]	− 0.067		− 0.116 [− 0.344, 0.112]	− 0.096		0.041 [− 0.191, 0.273]	0.034		− 0.137 [− 0.365, .091]	− 0.114		0.208 [− 0.017, 0.433]	0.172
Sex × AQ non-social traits		0.136 [− 0.098, 0.370]	0.115		0.188 [− 0.046, 0.422]	0.159		0.132 [− 0.107, 0.370]	0.111		0.014 [− 0.220, 0.249]	0.012		− 0.113 [− 0.344, 0.118]	− 0.095
AQ social traits × AQ non-social traits		0.067 [− 0.145, 0.279]	0.065		0.115 [− 0.097, 0.327]	0.111		0.029 [− 0.187, 0.245]	0.028		0.027 [− 0.187, 0.239]	0.026		− 0.044 [− 0.253, 0.166]	− 0.042
Sex × AQ social traits × AQ non-social traits		− 0.019 [− 0.269, 0.230]	− 0.016		− 0.090 [− 0.340, 0.159]	− 0.074		0.011 [− 0.243, 0.265]	0.009		0.025 [− 0.225, 0.275]	0.020		0.056 [− 0.190, 0.303]	0.046

*Sex* participants’ sex dummy coding (Males = 0; Females = 1), *ASI-3* anxiety sensitivity index, *BDI-II* Beck depression inventory, *AQ* autism spectrum quotient

*p < 0.05

**p < 0.01

***p < 0.001

The linear regression model for the prediction of cognitive concerns assessed by ASI-3 cognitive concerns subscale was significant, *F*(7,341) = 3.54; *p* = 0.001; *R* = 0.262; *R*^*2*^ = 0.069. Results showed that AQ social traits (*β* = 0.302; *t* = 3.16; *p* = 0.002) and sex (*β* = 0.114; *t* = 2.14; *p* = 0.033) were specific predictors of the measure.

The linear regression model for the prediction of social concerns assessed by ASI-3 social concerns subscale, *F*(7,341) = 3.28; *p* = 0.002; *R* = 0.253; *R*^*2*^ = 0.064, was significant. Results showed that AQ social traits (*β* = 0.342; *t* = 3.56; *p* < 0.001) was the only independent and specific predictor of the social concerns measure.

The linear regression model for the prediction of physical concerns assessed by ASI-3 physical concerns subscale was not significant, *F*(7,341) = 1.58; *p* = 0.135; *R* = 0.179; *R*^*2*^ = 0.032.

The linear regression model for the prediction of depression assessed by BDI-II total score, *F*(7,341) = 4.77; *p* < 0.001; *R* = 0.301; *R*^*2*^ = 0.090, was significant. However, no measure specifically predicted the depression measure.

Relevantly, no significant effect of AQ non-social traits or interactions was found in each regression model.

## Discussion

Results showed that social autistic traits and sex predicted ASI-3 total score indexing the degree to which an individual fears anxiety-related sensation based on the expectation that such sensations may have harmful consequences (“fear of fear”). The significant effect of sex in predicting general AS is consistent with data showing higher levels of AS in non-clinical females then in males (e.g., Jurin et al., 2012), although this sex difference has not been systematically found in literature (e.g., Osman et al., 2010). More interestingly, social autistic traits predicted AS domains related to cognitive (“I worry that I might be going crazy”) and social (“I worry that other people will notice my anxiety”) concerns, but not physical concerns (“It scares me when my heart beats rapidly”).

As regard depression symptoms, we did not find a predictive role of autistic traits on the BDI-II score. A tentative interpretation of this negative result could consider that previous studies showing increased depressive symptoms in individuals with high autistic traits mainly involved children, adolescents, and older adults (Pine et al., 2008; Scherff et al., 2014; Wallace et al., 2016) while very scarce data are available on young adults (Kanne et al., 2009), as the present sample. Moreover, some studies suggested that anxiety symptoms are particularly salient for autistic individuals, as they report severe levels of anxiety in comparison with moderate levels of depression symptoms (Park et al., 2020), and the strength of depressive symptoms seems related to the individual’s ability to regulate own emotional responses (Diefenbach et al., 2021). Thus, young adults could be able to deal with depressive symptoms better than anxiety symptoms.

The present results did not reveal any moderating effect of sex on social autistic traits in predicting AS, suggesting that there is no sex specificity in the relationship between social traits and anxiety sensitivity. Therefore, increased cognitive and social anxiety-related concerns in those with high social autistic traits could be considered a sex-independent phenotypic characteristic of adults with autism, reflecting sex-common social difficulties that are ubiquitous in individuals with autism (Baron-Cohen et al., 2015).

The present findings are the first to reveal a dissociable influence of social and non-social autistic traits on AS, with a specific role of social traits on AS and on its cognitive and social aspects. Social and non-social autistic traits are related to different cognitive processes, as social traits are characterized by difficulties in social interactions, communication and orienting towards social cues, while non-social traits are mainly associated to systemizing (Hoekstra et al., 2008; Warrier et al., 2019). Interestingly, Warrier et al. (2019) revealed genetic dissociations between social and non-social traits both in clinical and non-clinical autism, and identified shared genetics between the social traits and psychiatric conditions, but only limited shared genetics between non-social traits and psychiatric conditions. Consistently, the present findings indicate that people loading high on social autistic traits could be particularly vulnerable to develop psychopathological conditions, with specific concerns relating to fear of psychological dyscontrol or social threats. On the other hand, and again consistently with Warrier et al. (2019), we observed no relationship between non-social traits and both anxiety sensitivity and depression. Anxiety in individuals with autism has been associated to restricted and repetitive behaviours (Joyce et al., 2017; Rodgers et al., 2012), apparently at odds with both the present and Warrier et al.’s (2019) findings. Some authors suggested that anxiety can be related of fears of changing in routines in autism (Gillott et al., 2001), thus individuals with autism may engage in repetitive behaviours to relieve their anxiety and may become anxious because social environments are not organized to accommodate their repetitive behaviours (Zandt et al., 2006). In this respect, non-social autistic traits could be related to a highly specific kind of anxiety strongly connected to potential interference with routines. Here, we did not test this aspect of anxiety, but rather assessed self-related negative worries on anxiety signs, and results indicated that non-social autistic traits are not related in no way with fears and concerns on the potential negative consequences of anxiety-related symptoms and sensations.

The present findings are also consistent with research showing that the AQ, among other autism trait measures, shares variance with measures of more general personality traits (Ingersoll et al., 2011). Indeed, following the Big Five personality trait model, it has been demonstrated that social autistic traits are related to neuroticism and non-social traits to conscientiousness (Wakabayashi et al., 2006). Interestingly, moreover, a meta-analysis showed that neuroticism trait levels are related to severity of anxiety and depression (Kotov et al., 2010). For these reasons, future studies should consider general personality traits to clarify mechanisms underpinning specificities in the relationship between social and non-social autistic traits with psychopathologically relevant signs, as anxiety and depression.

Our results can have relevant clinical implications since AS plays an important role in the onset and the maintenance of several anxiety disorders (Calamari et al., 2008; Norton et al., 1997; Olthuis et al., 2014; White et al., 2006). In a recent neurofunctional interpretative model accounting for the frequent co-occurrence of autism and affective disorders, Burrows et al. (2017) suggested that difficulties with flexible switching of attention in response to distressing events, as well as heightened self-focused attention, can jointly predict increased tendency of perseverating on negative information about oneself, as in worry and rumination (McEvoy et al., 2010). Consistently, neurofunctional changes in structures involved in self-focused thoughts, as the midline cortical structures, can predict anxiety onset in participants with high autistic traits (Mikita et al., 2016). Increased appraisal on anxiety, as revealed by the higher ASI-3 total score, in persons with high social autistic traits support data on repetitive negative thinking in autism (Burrows et al., 2017), and further elucidate that the aspects defying the social autistic traits, i.e., reduced social skills, attention switching, communication and imagination, can make the individual more prone to get stuck on negative self-referential cognitions, in turn increasing the possibility to develop clinical anxiety. The process of perseverating on self-referential information represents an important marker of transdiagnostic vulnerability across different clinical groups (McEvoy et al., 2010; Treynor et al., 2003; Watkins, 2008). Our results showing that individuals with high social traits are concerned of psychological and social, but not somatic, anxiety symptoms underscore the importance of considering both the process and the content of self-referential appraisal for tailoring personalized treatment strategies. In this respect, the lack of concerns about somatic, interoceptive sensations in individuals with high social autistic traits could be related to altered processing of interoceptive information (Garfinkel et al., 2016; Soker-Elimaliah et al., 2020), although understanding mechanism underpinning relationships between interoceptive processing and specific domains of AS deserves direct investigation.

Finally, it’s important to note that in the present study we adopted the two-factor model of AQ (Hoekstra et al., 2008). However, different AQ factor models have been proposed. Recently, English et al. (2020) performed confirmatory factor analyses across competing factor models of the AQ and results strongly supported Russell-Smith et al.’s (2011) model differentiating three factors: social skill, details/patterns, and communication/mindreading. For these reasons, future studies should assess anxiety and depression symptoms relating to different autistic traits by relying upon AQ structures as the three-factor model (Russell-Smith et al., 2011).

In conclusion, the present results emphasize the need of assessing multiple domains of anxiety in studies on affective disorders in individuals on the autistic spectrum. Defining the pattern of AS domains relating to specific traits, as social autistic traits, can represent a useful approach for highlighting the individual’s vulnerability for developing specific psychopathological symptoms.
